# Opioid-sparing multimodal analgesia for post-craniotomy pain: a randomized, double-blind, placebo-controlled trial

**DOI:** 10.1186/s12871-025-03306-5

**Published:** 2025-08-29

**Authors:** Essamedin M. Negm, Mohammed A. Younus, Ahmed A. Morsy, Sahar M. S. El Gammal, Mona A. El-Harrisi, Fayrouz A. Abdel Sameaa, Rawan A. M. Rashad, Tamer S. Elserafy, Ahmed M. Gouda

**Affiliations:** 1 Department of Anaesthesia, ICU and Pain Management, Faculty of Medicine, Zagazig University, Zagazig, Egypt; 2 Department of Anaesthesia, ICU and Pain Management, Almogarif Hospital, Tripoli, Libya; 3 Department of Neurosurgery, Faculty of Medicine, Zagazig University, Zagazig, Egypt; 4 Zagazig University Hospitals, Zagazig, Egypt; 5 Department of Neurology, Faculty of Medicine, Zagazig University, Zagazig, Egypt; 6 Department of Pharmacy Practice, Faculty of Pharmacy, Zagazig University, Zagazig, Egypt

**Keywords:** Craniotomy, Postoperative pain, Multimodal analgesia (MMA), Opioid-sparing, Visual analog scale (VAS), Dexmedetomidine, Scalp block

## Abstract

**Background:**

Postoperative pain control in neurosurgical patients particularly after elective craniotomy remains clinically challenging due to the need for early neurological assessment and the adverse effects associated with opioid use. This study aimed to compare the efficacy and safety of an opioid-sparing multimodal analgesia (MMA) protocol versus a conventional opioid-based regimen for managing post-craniotomy pain.

**Methodology:**

This prospective, randomized controlled trial was conducted over 12 months at Zagazig University Hospitals and included 60 adult patients (aged 18–65 years, American Society of Anesthesiologists )ASA( physical status I–II) scheduled for elective supratentorial craniotomy with planned postoperative intensive care unit (ICU) admission. Patients were randomly assigned in a 1:1 ratio to either a multimodal opioid-sparing analgesia group (Group M, *n* = 30) or a conventional opioid-based analgesia group (Group O, *n* = 30) using simple randomization. The MMA protocol included preoperative oral gabapentin, intraoperative dexmedetomidine infusion, a postoperative scalp block with bupivacaine, and scheduled intravenous (IV) acetaminophen and ketorolac. The opioid group received scheduled IV morphine according to institutional practice. The primary outcome was the Visual Analog Scale (VAS) score at 2 h postoperatively. Secondary outcomes included time to first rescue analgesia, total opioid consumption, sedation scores, oxygen saturation, postoperative nausea and vomiting (PONV), and patient satisfaction.

**Results:**

VAS scores were significantly lower in Group M at 1, 2, and 4 h postoperatively (*P* = 0.046, 0.039, and 0.045, respectively). A highly significant difference in sedation scores was observed between the groups at 30 min, 1 h, and 4 h (*P* < 0.001). Additionally, Group M had a significantly lower frequency of vomiting (*P* = 0.034); however, excellent satisfaction scores were more frequently reported in Group O, despite the objectively superior analgesic and safety profile observed in Group M.

**Conclusion:**

In this randomized controlled trial, opioid-sparing MMA provided superior postoperative pain control after elective craniotomy, with fewer adverse effects compared to conventional opioid-based regimens. These results support the incorporation of MMA into standard postoperative protocols and align with the principles of Enhanced Recovery After Surgery (ERAS) in neurosurgical care.

**Trial registration:**

This trial is registered with ClinicalTrials.gov under the identifier NCT05474040, with the initial registration on 26 July 2022, and retrospective registration available at ClinicalTrials.gov.

**Supplementary Information:**

The online version contains supplementary material available at 10.1186/s12871-025-03306-5.

## Introduction

Postoperative pain is a common consequence of various neurosurgical and neuroradiological interventions, with approximately 60% of patients experiencing moderate-to-severe pain at the surgical site within the first 24 h post-craniotomy. This pain is predominantly superficial, suggesting a somatic rather than visceral origin, primarily arising from soft tissue and pericranial muscle trauma rather than brain tissue itself [1, 2]. Several factors, including gender, tumor size and type, surgical site, surgical technique, muscle resection, and steroid use, have been investigated for their association with the incidence and severity of postoperative pain [3].

Inadequate pain management can lead to serious complications such as hypertension, postoperative hematoma, elevated intracranial pressure (ICP), seizures, cerebral edema, and stroke [4]. For patients with impaired cerebral autoregulation, poorly controlled pain can further increase ICP, while arterial or intracranial hypertension raises the risk of intracranial hemorrhage [5]. Furthermore, effective postoperative pain control is crucial for maintaining optimal neurological assessment after craniotomy. Neurosurgeons rely on early and accurate neurological evaluations, and excessive sedation or opioid-related side effects can obscure clinical findings. Proper pain management strategies may also help minimize the need for additional diagnostic imaging, such as computed tomography (CT) scans, improving overall patient outcomes [6, 7]. The use of opioid analgesics in neurosurgical patients raises several concerns, including the risks of respiratory depression, hypercarbia, drowsiness, elevated ICP, and delayed ventilator weaning. Additionally, issues related to opioid dependence and the perception of opioids as a last-resort treatment have further limited their routine use [8]. Due to these challenges, the widespread use of opioids in postoperative neurosurgical pain management has been restricted, often leading to suboptimal analgesia [9]. These concerns have also prompted the incorporation of multimodal analgesia (MMA) protocols into Enhanced Recovery After Surgery (ERAS) pathways in neurosurgical settings. This dilemma, combined with an increasing focus on opioid-free anesthesia and perioperative analgesia, has driven interest in alternative pain management strategies [10].

Effective postoperative pain management in patients undergoing craniotomy remains a clinical challenge. Unlike other neurosurgical procedures such as spinal surgery, craniotomy presents unique considerations, including the need for early neurological assessment and concerns about sedation and ICP. Although craniotomy was historically believed to be associated with minimal postoperative pain, recent evidence has challenged this assumption, particularly in supratentorial cases. Poorly controlled pain in this context may delay recovery, increase ICP, and reduce patient satisfaction. Current strategies often rely heavily on opioids, which are associated with significant adverse effects, prompting a growing interest in MMA protocols that reduce opioid use while improving pain outcomes. However, high-quality evidence supporting MMA in elective craniotomy remains limited, particularly in protocols that combine multiple agents in a standardized approach. MMA involves the combination of different classes of analgesic agents with diverse mechanisms of action and distinct adverse effect profiles. This strategy enhances synergistic analgesic effects while simultaneously reducing opioid consumption, making it a potentially optimal and effective option for postoperative pain control. However, there remains a lack of consensus regarding the use of common systemic analgesics for post-craniotomy pain. While the benefits of MMA are well-established in general surgery, high-quality evidence supporting its use in elective craniotomy remains limited. The current study addresses this gap by implementing a standardized, multimodal regimen tailored to the specific needs of neurosurgical patients. Various analgesic adjuvants, including paracetamol, non-steroidal anti-inflammatory drugs (NSAIDs), gabapentin, dexmedetomidine, and scalp blocks, can be used individually or in combination, yet further evidence is needed to establish standardized guidelines for their application [11]. This study was designed to compare two comprehensive and clinically relevant strategies for managing post-craniotomy pain: a conventional opioid-based analgesic protocol and a multimodal, opioid-sparing regimen incorporating systemic agents alongside a regional scalp block. Rather than isolating the effects of individual components, this study evaluated the overall effectiveness of two comprehensive strategies. The multimodal protocol was designed to reflect real-world ERAS implementation by integrating multiple agents into a standardized regime. The primary objectives were to assess the adequacy of pain relief through the Visual Analog Scale (VAS) at 2 h postoperatively, the time to first rescue analgesia, and the total opioid consumption within the first 24 h. Secondary objectives included evaluating the incidence of adverse effects such as postoperative nausea and vomiting (PONV), sedation levels, and overall patient satisfaction associated with each analgesic regimen. We hypothesized that patients receiving a multimodal opioid-sparing analgesic regimen would report significantly lower postoperative pain scores at 2 h compared to those receiving conventional opioid-based analgesia.

## Patients and methods

### Study design and setting

This prospective, randomized, controlled clinical trial was conducted over a 12-month period, from December 2022 to December 2023, at Zagazig University Hospitals. The study was designed and reported in accordance with the CONSORT guidelines for randomized controlled trials. Postoperative ICU patients who had undergone elective craniotomy, all performed by a single neurosurgeon, were enrolled. The research was implemented in both the operating room and the postoperative surgical ICU, under standardized conditions to ensure consistency in patient care and data collection.

### Ethical approval and trial registration

This study received ethical approval from the Institutional Review Board (IRB) of the Faculty of Medicine, Zagazig University (IRB number 8099-12-10-2021) on October 12, 2021. Patient enrollment began in December 2022. The trial was registered at ClinicalTrials.gov (NCT05474040) on July 22, 2022, prior to enrollment. The protocol and outcomes remained unchanged. The study was conducted in strict adherence to the ethical principles outlined in the Declaration of Helsinki. Informed consent was obtained from all patients or their legally authorized representatives prior to their inclusion in the study. The consent process ensured participants received comprehensive information about the study’s purpose, procedures, potential risks, and benefits, allowing them to make an informed decision.​.

All patients were informed of their right to withdraw from the study at any time without consequences. Confidentiality was strictly maintained, and all collected data were used solely for scientific purposes. Prior to surgery, each patient underwent a comprehensive preoperative interview, during which the study’s objectives and endpoints were thoroughly explained. Additionally, patients received a detailed explanation of the VAS for pain assessment to ensure accurate self-reporting of pain levels.

### Inclusion criteria

Patients were eligible for inclusion if they were adults aged 18 to 65 years and classified as American Society of Anesthesiologists (ASA) physical status I or II. All participants were scheduled for elective craniotomy under general anesthesia and were capable of oral medication intake. Patients receiving short-term steroid therapy (< 5 days) or antiepileptic treatment for ≤ 2 weeks prior to surgery were eligible for inclusion. However, patients receiving systemic corticosteroids for ≥ 5 consecutive days or chronic antiepileptic therapy exceeding 2 weeks were excluded, as prolonged use could influence pain perception and introduce confounding variables.

### Exclusion criteria

Patients were excluded if they had neurological impairment, including a Glasgow Coma Scale (GCS) score below 14, or medical conditions such as uncontrolled hypertension, renal or hepatic dysfunction, or bronchial asthma. Those with a history of hypersensitivity to any of the medications used in the study were also excluded.

Surgical factors leading to exclusion included extensive procedures lasting more than six hours, cases complicated by massive intracranial hemorrhage, or those requiring postoperative ventilatory support. patients receiving long-term therapy with analgesics, anticonvulsants, neuropathic agents, antidepressants, or systemic corticosteroids, as well as individuals with a history of drug dependence, were also ineligible. Additionally, patients with psychiatric disorders requiring pharmacologic treatment and those with a history of previous craniotomy were excluded to minimize confounding variables. All inclusion and exclusion criteria were assessed during the preoperative evaluation prior to randomization to ensure eligibility and consistency.

### Sample size calculation

The sample size was determined based on data from a previous study by Dilmen et al. (2016) [12], which evaluated MMA after craniotomy and reported a between-group difference in VAS score of approximately 1.3 points. This exceeds the minimal clinically important difference (MCID) of 1.0 for postoperative pain, making it both statistically and clinically meaningful in the neurosurgical context. Using the G*Power software (version 3.1.9.4), we calculated the required sample size with an effect size of 0.79, assuming a standard deviation of 1.25, a two-tailed alpha error (α) of 0.05, and a power of 80%. Based on these parameters, the minimum required sample size was determined to be 27 patients per group. We included 30 patients per group to account for potential dropouts or exclusions (Supplementary Fig. 1).

### Method of randomization

Following informed consent, patients were prospectively randomized in a 1:1 ratio, with the individual patient as the unit of randomization. Simple randomization was applied using a computer-generated random number sequence, which was managed by an independent research coordinator. Allocation concealment was ensured through the use of sealed, opaque, sequentially numbered envelopes, prepared in advance by the coordinator and opened only after patient enrollment. Although simple randomization does not inherently guarantee equal group sizes particularly in smaller trials the process resulted in balanced groups in our study (30 patients per group). In future studies, block or stratified randomization may be considered to optimize group balance where sample size is more limited.

### Study groups

The 60 enrolled patients were randomly assigned to one of two treatment groups, with allocation conducted in a blind manner. Group M (Multimodal Opioid-Sparing Analgesia): This group comprised 30 patients who received a multimodal opioid-sparing analgesia regimen, as detailed in previous studies [13–17] (Supplementary Table 1). Group O (Opioid Analgesia): The remaining 30 patients were assigned to the opioid-based analgesia group, receiving postoperative pain management in accordance with the standard Zagazig University protocol. This regimen consisted of intravenous (IV) morphine (2.5 mg) every 6 h, initiated once patients were alert enough to report pain [6].

### Rescue analgesia protocol

For both groups, rescue analgesia was administered in cases of inadequate pain relief, consisting of an additional 2 mg of IV morphine as needed. Inadequate pain relief was defined as a VAS score ≥ 4, which was the standardized threshold across both study arms. The rescue dose and timing were consistent: 2 mg of IV morphine given immediately upon reaching the threshold. The time to first rescue dose was recorded as a secondary outcome. Additionally, for outcome interpretation, effective pain relief was defined as maintaining a VAS score < 4 without requiring rescue analgesia during the postoperative period.

### Patient Preparation

#### Preoperative evaluation

A standardized preoperative assessment was conducted for all patients. This included a comprehensive history covering demographics, current complaints, comorbidities, medication use, and previous surgeries. Clinical examination involved vital signs, body mass index (BMI), neurological status, and electrocardiography (ECG) to determine anesthesia fitness. Routine laboratory investigations complete blood count (CBC), liver and renal function, blood glucose, and coagulation profile were obtained. As required by the institutional infection control policy, screening for viral infections including human immunodeficiency virus (HIV), hepatitis C virus antibodies (HCV Ab), and hepatitis B surface antigen (HBV Ag) was conducted for patients undergoing invasive procedures such as central venous catheter placement or scalp block. block. Additional tests were requested when clinically indicated.

#### Radiological investigations

Preoperative imaging included CT and/or magnetic resonance imaging (MRI) of the brain, selected based on the surgical indication.

#### Anesthetic consultation

All patients underwent a preoperative evaluation by the anesthesia team, during which ASA classification was determined [18]. Patients were also educated on how to assess and communicate postoperative pain using VAS, where 0 represents no pain and 10 indicates the worst possible pain [19, 20] (Supplementary Fig. 2).

### Operative room setup

#### Preoperative medication and sedation

In the multimodal (M) group, gabapentin was administered at a dose of 600 mg the night before surgery and again two hours before anesthesia induction. Additionally, IV midazolam (0.05 mg/kg) was administered for sedation in the anesthesia preparation room prior to surgery.

#### Intraoperative monitoring

Upon arrival in the operating room, comprehensive hemodynamic monitoring was initiated, including heart rate (HR), noninvasive and invasive blood pressure (BP), ECG, and pulse oximetry to ensure patient stability throughout the procedure.

### Central Venous Line (CVL) placement

As per the institutional protocol, central venous catheterization was performed one day before surgery in the ICU. A mandatory X-ray confirmation was required before surgery to verify correct catheter placement and exclude any complications.

### Anesthetic technique (Standard Zagazig university Protocol)

#### Induction and maintenance of anesthesia

All surgical procedures were conducted under general anesthesia. Patients underwent pre-oxygenation with 100% oxygen for three minutes prior to induction. IV fentanyl (1–2 µg/kg) was administered 2–3 min before induction to provide analgesia. Anesthesia was induced with propofol (0.5–2 mg/kg), titrated according to clinical response and hemodynamic stability.

For tracheal intubation, a cuffed endotracheal tube of appropriate size was used, facilitated by atracurium (0.5 mg/kg). To maintain muscle relaxation, incremental doses of atracurium (0.1 mg/kg) were administered every 20–30 min throughout surgery. Train-of-four (TOF) peripheral nerve stimulation was employed to guide neuromuscular blocking agent (NMBA) dosing and assess the depth of neuromuscular blockade. In patients with localized upper motor neuron lesions (e.g., due to mass effect) but preserved consciousness and neurological function, TOF monitoring was applied to the unaffected limb to ensure accurate assessment. Patients with significant neurological impairment such as altered consciousness, hemiparesis, or GCS < 14 were excluded from the study. Depth of anesthesia was guided by standard clinical parameters and TOF monitoring, as BIS and SedLine monitors were not available at our institution during the study period.

### Summary of interventions per study group

To improve clarity and ensure reproducibility, the anesthesia and analgesia protocols for each study group are summarized below.

#### Group M (Multimodal Analgesia Group)

General anesthesia was induced with IV propofol, fentanyl, and atracurium, and maintained with isoflurane in an oxygen–air mixture. Dexmedetomidine infusion was administered intraoperatively (loading dose: 1 µg/kg over 10 min, followed by 0.5 µg/kg/h). Oral gabapentin (600 mg) was administered the night before surgery and repeated 2 h preoperatively. Postoperative analgesia included IV acetaminophen (10–15 mg/kg every 8 h) and ketorolac (15–30 mg every 6 h). A bilateral scalp block was performed at the end of surgery using 0.25% bupivacaine (total volume: 20 mL) targeting the major sensory nerves of the scalp.

#### Group O (Opioid-Based Analgesia Group)

General anesthesia was identical to Group M but without dexmedetomidine. Postoperative analgesia consisted of scheduled IV morphine (2.5 mg every 6 h), with additional rescue doses as needed. To maintain blinding, patients received placebo oral tablets and a simulated scalp block using 1% lidocaine (2 mL per injection site), which provided no lasting analgesic effect.

#### Additional interventions for the multimodal group (M Group)

For patients in the M group, additional intraoperative interventions were implemented for research purposes. Dexmedetomidine infusion was initiated 15 min after intubation, with a loading dose of 1 µg/kg, diluted to 20 mL and administered over 10 min, followed by a continuous IV infusion at a rate of 0.5 µg/kg/hr via an infusion pump until skin closure.

### Ventilation and anesthetic maintenance

Oxygen (40% in air mixture) was administered via volume-controlled ventilation, with minute ventilation adjusted to maintain end-tidal CO₂ (ETCO₂) between 30 and 35 mmHg. Anesthesia was maintained with isoflurane (1–2%), titrated based on patient response and hemodynamic stability.

### Intraoperative monitoring and hemodynamic management

Continuous hemodynamic monitoring was performed throughout surgery. Any intraoperative hemodynamic instability was promptly managed to ensure adequate cerebral perfusion pressure (CPP). BP was maintained to keep CPP within 65–80 mmHg. Assuming normal ICP or central venous pressure (CVP) (5–10 mmHg), the mean arterial pressure (MAP) was kept within 75–90 mmHg for uncomplicated patients. Given individual variations, cerebral blood flow (CBF) autoregulation typically occurs within a MAP range of 60–150 mmHg.

Fluid management was optimized to maintain normovolemia, ensuring adequate cerebral perfusion while preventing cerebral edema. Anesthetic agents were titrated according to the surgical stimulus, balancing the need to prevent hypotensive anesthetic effects while mitigating hypertensive responses to stimulation. Hypertension was managed primarily by deepening anesthesia. If necessary, short-acting vasodilator boluses or continuous vasodilator infusion (e.g., nitroglycerin 10–400 mcg/min IV) were administered. Conversely, vasoconstrictors were used to counteract anesthetic-induced vasodilation, with small boluses of short-acting agents (e.g., ephedrine 5–10 mg IV) given for hypotensive episodes.

At the end of surgery, isoflurane was discontinued, and neuromuscular blockade was reversed using neostigmine (0.04 mg/kg) and atropine (0.02 mg/kg).

### Scalp block technique and timing

To ensure targeted postoperative analgesia while minimizing intraoperative confounding, the scalp block was administered at the end of surgery after skin closure and prior to extubation. This timing confined the analgesic effect to the postoperative period, avoided interference with intraoperative hemodynamics, and preserved blinding of the surgical and ICU teams. In the multimodal group (Group M), a landmark-based bilateral scalp block was performed using a 23G short-bevel needle, targeting six sensory nerves: supratrochlear, supraorbital, zygomaticotemporal, auriculotemporal, greater occipital, and lesser occipital. Each injection site received 2–4 mL of 0.25% bupivacaine, totaling approximately 20 mL.

In the control group (Group O), a simulated block was conducted using 2 mL of 1% lidocaine at the same anatomical landmarks. Given lidocaine’s short duration of action, this approach ensured sensory masking for effective blinding without providing prolonged analgesia during the postoperative assessment period. t.

#### Post-operative care

Postoperatively, all patients were extubated within 30–45 min, either in the operating room or shortly after admission to the ICU, depending on their neurological and hemodynamic stability. In some cases, patients were transferred to the ICU under anesthesia to allow for controlled emergence and safe extubation, but no patient remained intubated beyond this period. Patients requiring prolonged postoperative ventilation (> 1 h) or those with delayed extubation due to sedation or respiratory compromise were excluded from the study. Pain, sedation, and PONV assessments began only after extubation, ensuring uniformity in the timing of outcome measurements across both groups. Pain assessment was conducted using VAS [19] at predefined time points: 30 min, 1, 2, 4, 8, 16, and 24 h postoperatively, with the zero point being the time of emergence from anesthesia and endotracheal extubation. An observer blinded to treatment allocation (one of the researchers) performed these assessments.

#### Postoperative analgesia protocol

In the multimodal group (Group M), patients received oral gabapentin 600 mg both the night before surgery and again two hours prior to induction. Intraoperatively, dexmedetomidine was administered at a loading dose of 1 µg/kg over 10 min followed by a continuous infusion of 0.5 µg/kg/h. After surgery, scheduled analgesics included IV acetaminophen (10–15 mg/kg every 8 h) and IV ketorolac (15–30 mg every 6 h). A scalp block was performed at the end of surgery using 0.25% bupivacaine, with 2 mL injected at each site targeting six bilateral sensory nerves. Rescue analgesia was provided with IV morphine 2 mg when the VAS score reached ≥ 4. In the opioid group (Group O), postoperative analgesia consisted of scheduled IV morphine 2.5 mg every 6 h, and the same rescue protocol was followed using IV morphine 2 mg if VAS ≥ 4. In both groups, the time to first rescue analgesia was recorded, and total opioid consumption was calculated over the first 24 postoperative hours.

Autonomic cardiovascular responses to pain, including HR and MAP, were recorded at 1 and 2 h postoperatively, followed by every 2 h during the first 24 h, including intraoperative values. Oxygen saturation (SpO₂) was measured on room air at scheduled time points over 24 h.

Sedation levels were evaluated using a four-point scale at 30 min, 1, 2, 4, 8, 16, and 24 h postoperatively, with scores ranging from 1 (awake and communicative) to 4 (deeply sedated) [21]. The severity of PONV was assessed using a four-point (0–3) scoring system, where 0 = no nausea or retching, 1 = mild nausea/retching, 2 = vomiting one or two times in 30 min, and 3 = vomiting more than twice in 30 min [22]. At 24 h post-zero point, patients were asked to rate their overall satisfaction with analgesia on a 1–3 verbal scale, where 1 = unsatisfactory, 2 = satisfactory, and 3 = excellent analgesia [23].

### Blinding of the study

The anesthesia researcher (RA) was responsible for obtaining informed consent, explaining the surgical procedure, and outlining the study objectives to participants. To ensure proper blinding across both groups, the following measures were implemented. In the opioid group (Group O), a simulated scalp block was performed using 1% lidocaine (2 mL per injection site) to mimic the procedure without providing prolonged analgesia. In contrast, the multimodal group (Group M) received an actual scalp block using 0.25% bupivacaine. For the dexmedetomidine infusion, both the active drug (in Group M) and the placebo (in Group O) were prepared in identical syringes with neutral labeling by an independent pharmacist not involved in patient care. This maintained blinding for anesthesiologists, surgical staff, ICU personnel, and patients. In both groups, placebo administrations were used to match the timing and route of active interventions: in Group M, patients received IV and oral placebos at the time morphine would have been administered; in Group O, patients received placebo infusions and oral placebo capsules alongside their opioid regimen. The ICU data collection team responsible for VAS assessments, opioid dose recording, and monitoring adverse effects was blinded to group assignments. Additionally, the anesthesia and ICU teams did not participate in data analysis to further minimize potential bias.

### Study outcomes

The primary outcome was the VAS pain score at 2 h postoperatively, which served as the basis for hypothesis testing and sample size calculation. A difference of ≥ 1.0 point was considered clinically meaningful. Secondary outcomes included time to first rescue analgesia, total opioid consumption within 24 h, rescue morphine dose (VAS ≥ 4), sedation scores, oxygen saturation, patient satisfaction, and adverse effects such as PONV, pruritus, and respiratory depression. In Group M, total opioid use refers to rescue morphine only, while in Group O, it includes both scheduled and rescue doses. Inferential statistical testing was applied exclusively to the primary outcome (VAS at 2 h). Secondary outcomes were analyzed descriptively or using exploratory statistical comparisons, without correction for multiple testing.

### Statistical analysis

All collected data were coded, entered, and analyzed using SPSS version 20.0. Descriptive statistics were presented as mean ± standard deviation (SD) for continuous variables with normal distribution, and as median with interquartile range (IQR) for skewed or ordinal variables. Categorical variables were summarized using frequencies and percentages. The normality of data distribution was assessed using the Shapiro–Wilk test. According to data distribution, comparisons between groups were performed using the independent samples t-test for normally distributed continuous variables, and the Mann–Whitney U test for non-normally distributed or ordinal variables (e.g., sedation scores). Categorical data were analyzed using the Chi-square test or Fisher’s exact test when appropriate. We hypothesized that patients receiving MMA (Group M) would demonstrate a significantly lower VAS score at 2 h postoperatively compared to those receiving conventional opioid-based analgesia (Group O). This hypothesis served as the foundation for sample size estimation and statistical analysis. All other outcomes (e.g., time to first rescue analgesia, total opioid consumption, sedation levels, oxygen saturation, and patient satisfaction) were treated as secondary endpoints and analyzed descriptively. No formal correction for multiple comparisons (e.g., Bonferroni adjustment) was applied; thus, secondary outcome analyses should be interpreted as exploratory. A per-protocol analysis was conducted, including only patients who received the allocated intervention as planned. No participants were lost to follow-up, and no missing data were recorded during the 24-hour postoperative period. Statistical significance was defined as a p-value ≤ 0.05.

## Results

The VAS score at 2 h was the sole predefined primary outcome and is presented first, followed by exploratory analyses of secondary outcomes. Effect sizes and statistical comparisons are reported accordingly.

### Demographic and baseline characteristics

**Fig. 1:**
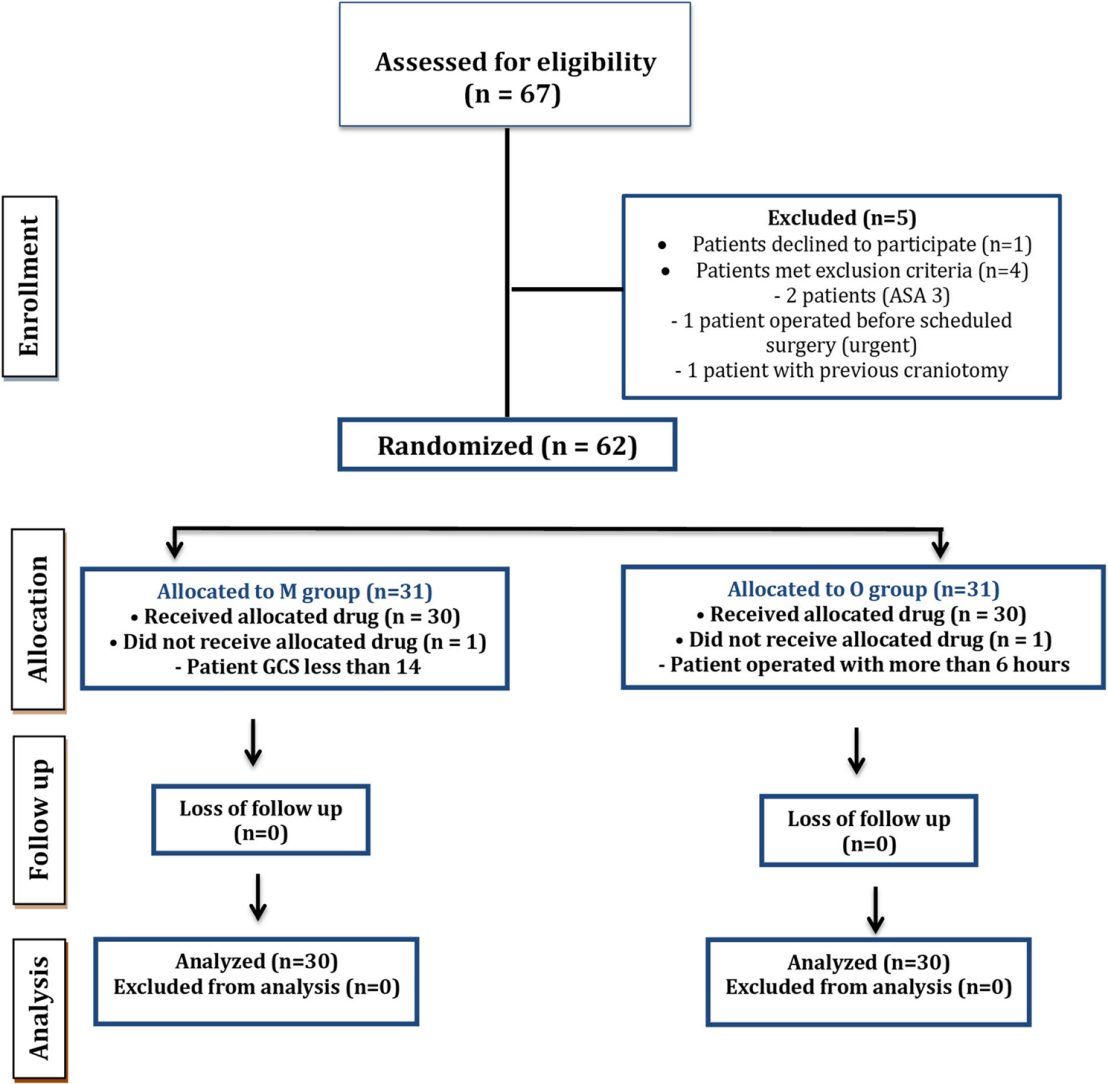
Enrollment flow chart of randomized patients

A total of 60 patients were included in the final analysis, selected from an initial cohort of 67, as detailed in Fig. 1. Baseline demographic data are presented in Table 1. There were no statistically significant differences between the groups in age, BMI, ASA physical status, duration of surgery, or surgical indication. These findings indicate successful randomization and baseline comparability between the two groups.

**Table 1:** Preoperative data of the two study groups

Variable	Group M (*n* = 30)	Group O (*n* = 30)	*P* value
Age (years)	41.37 ± 11.52 (95% CI: 37.3–45.4)	45.97 ± 13.77 (95% CI: 40.9–51.1)	0.166
**Gender**			
Male	18 (60%)	14 (46.7%)	0.301
Female	12 (40%)	16 (53.3%)	
BMI (kg/m²)	25.45 ± 2.62 (95% CI: 24.5–26.4)	25.31 ± 3.01 (95% CI: 24.2–26.5)	0.854
**Medical history and comorbidities**			
Smoking	7 (23.3%)	5 (16.7%)	0.519
Hypertension (HTN)	9 (30%)	7 (23.3%)	0.559
Diabetes Mellitus (DM)	8 (26.7%)	10 (33.3%)	0.573
Hepatitis C Virus (HCV)	2 (6.7%)	5 (16.7%)	0.228
Dyslipidemia	1 (3.3%)	2 (6.7%)	0.554
Thyroid disorders	1 (3.3%)	0 (0%)	0.313
Ischemic Heart Disease (IHD)	2 (6.7%)	1 (3.3%)	0.426
**Medication**			
Antihypertensives	9 (30%)	7 (23.3%)	0.559
Oral hypoglycemics	5 (16.7%)	4 (13.4%)	0.132
Insulin	3 (10%)	6 (20%)	0.243
L-thyroxine	1 (3.3%)	0 (0%)	0.313
Antiplatelets	5 (16.7%)	3 (10%)	0.143
Antiepileptics	7 (23.3%)	8 (26.7%)	0.568
Dexamethasone	24 (80%)	26 (86.7%)	0.863
**Clinical symptoms**			
Headache	25 (83.3%)	24 (80%)	0.739
Blurring of vision	21 (70%)	20 (66.7%)	0.781
Motor weakness	9 (30%)	12 (40%)	0.417
Sensory dysfunction	8 (26.7%)	8 (26.7%)	1.000
Seizures	7 (23.3%)	8 (26.7%)	0.766
Gait disturbances	1 (3.3%)	3 (10%)	0.426
Speech dysfunction	5 (16.7%)	6 (20%)	0.754
Lack of sphincter control	3 (10%)	4 (13.4%)	0.352
Mild confusion (sleepy)	3 (10%)	2 (6.7%)	0.564
**Surgical Data**			
ASA I	15 (50%)	14 (46.7%)	0.796
ASA II	15 (50%)	16 (53.3%)	
**Indication for Surgery**			
Supratentorial intra-axial SOL	9 (30%)	12 (40%)	0.531
Supratentorial extra-axial SOL	8 (26.7%)	9 (30%)	
Posterior fossa SOL	5 (16.7%)	3 (10%)	
Brain abscess	2 (6.6%)	2 (6.6%)	
Cerebral aneurysm clipping	2 (6.6%)	1 (3.4%)	
Resective epilepsy surgery	4 (13.4%)	3 (10%)	
Duration of surgery (min)	217.20 ± 59.83 (95% CI: 195.5–239.0)	232.33 ± 56.72 (95% CI: 211.9–252.8)	0.807

Continuous variables are presented as mean ± standard deviation (SD) with 95% confidence intervals (CI), and categorical variables are expressed as number (percentage)

*HTN* Hypertension, *DM* Diabetes Mellitus, *HCV* Hepatitis C Virus, *IHD* Ischemic Heart Disease, *ASA* American Society of Anesthesiologists, *SOL* Space-Occupying Lesion

### Primary outcome – pain intensity (VAS Score)

**Fig. 2:**
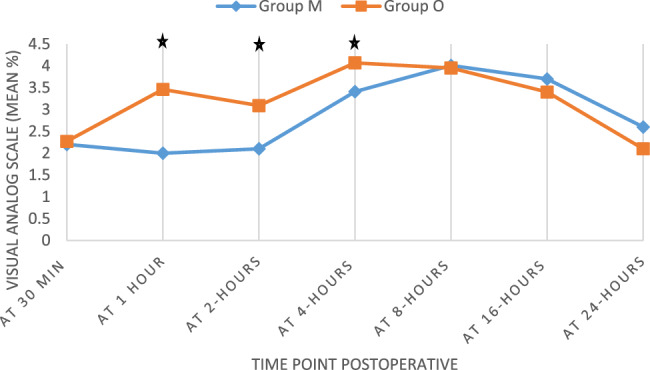
The visual analog scale (VAS) in the two groups studied at different times. ★ *p*-value < 0.05

The primary outcome was the VAS score at 2 h postoperatively. Group M reported significantly lower VAS scores (2.10 ± 0.69) compared to Group O (3.09 ± 0.68), with a mean difference of − 0.99 (*p* = 0.039), indicating superior early analgesia in the multimodal group. Additional VAS comparisons at other time points were exploratory: Group M demonstrated lower scores at 1 h (mean difference: − 1.46; *p* = 0.046) and 4 h (mean difference: − 0.66; *p* = 0.045). No significant differences were observed at 30 min, 8, 16, or 24 h (Table 2; Fig. 2).

**Table 2:** Visual analog scale (VAS) in the two groups studied at different times

Time Point	Group M (Mean ± SD) [95% CI]	Group O (Mean ± SD) [95% CI]	*P* value
30 min postoperative	2.20 ± 0.41 [2.05–2.35]	2.27 ± 0.45 [2.10–2.44]	0.549
1 h postoperative	2.00 ± 0.72 [1.73–2.27]	3.46 ± 0.50 [3.27–3.65]	0.046
2 h postoperative	2.10 ± 0.69 [1.84–2.36]	3.09 ± 0.68 [2.84–3.34]	0.039
4 h postoperative	3.41 ± 0.74 [3.13–3.69]	4.07 ± 0.81 [3.77–4.37]	0.045
8 h postoperative	4.01 ± 0.64 [3.77–4.25]	3.95 ± 0.89 [3.62–4.28]	0.521
16 h postoperative	3.70 ± 1.02 [3.32–4.08]	3.40 ± 0.87 [3.08–3.72]	0.519
24 h postoperative	2.60 ± 0.35 [2.47–2.73]	2.10 ± 0.69 [1.84–2.36]	0.552

*VAS* Visual Analog Scale, Data are presented as Mean ± Standard Deviation with 95% Confidence Interval

### Secondary outcomes – rescue analgesia and opioid consumption

Patients in Group M experienced more prolonged analgesia, as indicated by a significantly longer median time to first rescue analgesia (265.4 min vs. 191.4 min), with a median difference of approximately 74 min (*p* < 0.001). Group M also had significantly lower total opioid consumption (median: 2 mg [IQR: 2–6]) compared to Group O (median: 11 mg [IQR: 10–14]; *p* < 0.001). Notably, although the rescue morphine dose in Group M (2 mg [IQR: 2–6]) was significantly higher than in Group O (1 mg [IQR: 0–4]; *p* = 0.038), this reflects the study design: Group M received morphine only as rescue analgesia, while Group O received both scheduled and rescue morphine. These outcomes collectively underscore the opioid-sparing effect and prolonged analgesic duration of the multimodal regimen (Table 3, Supplementary Fig. 3 and Supplementary Fig. 4).

**Table 3:** Analgesia-related data in the two study groups

Variable	Group M (*n* = 30)	Group O (*n* = 30)	*P* value
Rescue analgesia			
No	18 (60%)	21 (70%)	0.600
Yes	12 (40%)	9 (30%)	
Time to first rescue analgesia (min)	265.43 ± 17.62 (95% CI: 258.4–272.5)	191.40 ± 16.10 (95% CI: 185.0–197.8)	< 0.001
Morphine dose consumption (rescue) (mg)	2 [2–6]	1 [0–4]	0.038
Total opioid dose (mg)	2 [2–6]	11 [10–14]	< 0.001

Continuous variables are presented as mean ± standard deviation (SD) with 95% confidence intervals (CI), or as median [interquartile range] where applicable. Categorical variables are presented as number (percentage). “Morphine dose consumption (rescue)” refers to the total amount of rescue morphine administered when VAS ≥ 4. “Total opioid dose” includes both scheduled morphine (2.5 mg every 6 h) and rescue morphine in Group O, and rescue-only morphine in Group M

### Sedation scores

Patients in Group O consistently exhibited higher sedation levels across all postoperative time points compared to Group M. At 30 min, the median sedation score was 2 [IQR: 2–3] in Group M versus 3 [IQR: 3–4] in Group O (*p* < 0.001). Comparable statistically significant differences were observed at 1 h (2 [1–3] vs. 3 [3]– [4], *p* < 0.001) and 4 h (2 [2]– [3] vs. 4 [3]– [4], *p* < 0.001). At 2, 8, 16, and 24 h, the differences remained significant, with consistently lower sedation scores in Group M (2 [1–3] vs. 3 [2–4], *p* = 0.014; 2 [1–3] vs. 3 [2–4], *p* = 0.020; 2 [1]– [2] vs. 3 [2–4], *p* = 0.023; and 2 [1]– [2] vs. 3 [2–4], *p* = 0.001, respectively). These findings indicate a consistently lower sedation burden in the multimodal group, which can be attributed to the avoidance of systemic opioids (Table 4). These findings, which indicate a consistently lower sedation burden in the multimodal group, can likely be attributed to the avoidance of systemic opioids.

**Table 4:** Sedation score, post operative nausea and vomiting)PONV(, other side effects, and patients’ satisfaction in the two studied groups

Sedation score	Group M (*n* = 30)	Group O (*n* = 30)				*P* value
At 30 min postoperative	2 [2–3]	3 [3–4]				< 0.001
At 1 h postoperative	2 [1–3]	3 [3–4]				< 0.001
At 2-hours postoperative	2 [1–3]	3 [2–4]				0.014
At 4-hours postoperative	2 [2–3]	4 [3–4]				< 0.001
At 8-hours postoperative	2 [1–3]	3 [2–4]				0.020
At 16-hours postoperative	2 [1–2]	3 [2–4]				0.023
At 24-hours postoperative	2 [1–2]	3 [2–4]				0.001
**PONV**						
No nausea or vomiting	22 (73.3%)		18 (60%)		0.034	
Sickness and retching	5 (16.7%)		6 (20%)			
Vomiting 1–2 times	3 (10%)		4 (13.3%)			
Vomiting 3 times	0 (0%)		2 (6.7%)			
**Other side effects**						
Pruritus	0(0%)		3(10%)		0.237	
Respiratory depressed	0(0%)		3(10%)		0.237	
Bronchospasm	0(0%)		2(6.7%)		0.492	
**Patients satisfaction**						
Unsatisfactory analgesia	6 (20%)			4 (13.3%)		0.028
Satisfactory analgesia	20 (66.7%)			15 (50%)		
Excellent analgesia	4 (13.3%)			11 (36.7%)		

*PONV* post operative nausea and vomiting

*S* Significant (*p* ≤ 0.05); HS: Highly significant (*p* ≤ 0.001); NS: Non-significant (*p* > 0.05). Sedation scores are represented as median [interquartile range]. Other categorical data are presented as number (percentage)

### Oxygen saturation

**Fig. 3:**
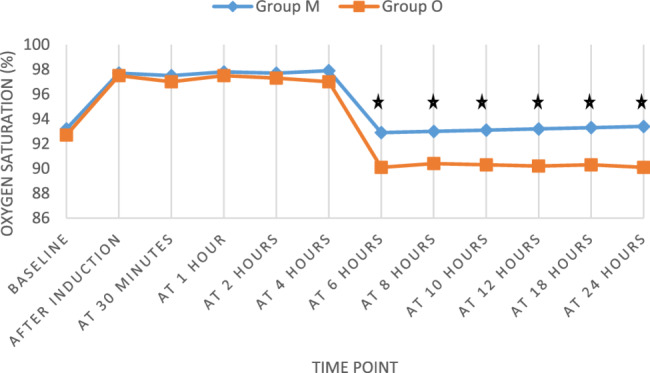
Oxygen saturation (%) of the study groups along the duration of follow-up. ★ *p*-value < 0.05

Oxygen saturation levels were comparable between groups at baseline and early postoperative hours (30 min, 1, 2, 4 h). However, Group M maintained significantly higher SpO₂ readings at 6, 8, 10, 12, 18, and 24 h post-op (*p* < 0.05) (Fig. 3).

### Hemodynamic parameters

**Fig. 4:**
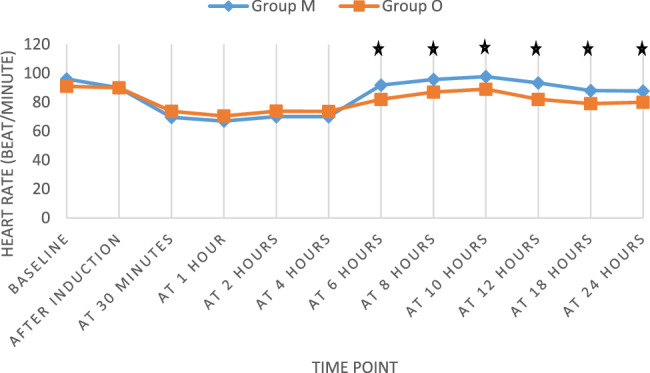
Heart rate (beat/minute) of the study groups along the duration of follow-up. ★ *p*-value < 0.05

**Fig. 5:**
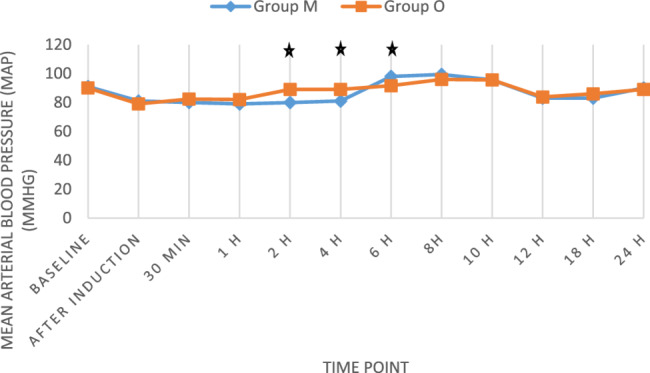
Mean arterial Blood pressure (MAP) (mmHg) of the study groups along the duration of follow-up. ★ *p*-value < 0.05

HR and MAP were monitored over 24 h. HR was significantly higher in Group M at 6, 8, 10, 12, 18, and 24 h (*p* < 0.05) (Fig. 4). MAP was significantly lower in Group M at 2, 4, and 6 h (*p* < 0.05), but comparable at other time points (Fig. 5). These findings may reflect the sympatholytic effect of dexmedetomidine in Group M.

### Postoperative nausea and vomiting and adverse effects

Group M had a lower incidence of PONV, with 73.3% of patients reporting no symptoms compared to 60% in Group O (*p* = 0.034). Although individual episodes of retching and vomiting were more frequent in Group O, these differences were not statistically significant. Adverse effects such as pruritus, respiratory depression, and bronchospasm were observed only in Group O (6.7–10%) but did not reach statistical significance (Table 4).

### Patient satisfaction

Despite better objective analgesia in Group M, patient satisfaction was paradoxically higher in Group O. Specifically, 36.7% of patients in Group O reported “excellent” analgesia compared to 13.3% in Group M (*p* = 0.028). This may reflect patients’ subjective perception of stronger pain relief with opioids, despite higher sedation and adverse effects (Table 4).

## Discussion

This randomized controlled trial demonstrated that a multimodal, opioid-sparing analgesic protocol significantly improved postoperative pain outcomes compared to conventional opioid-based analgesia in patients undergoing elective craniotomy. Specifically, patients in the multimodal group reported lower VAS pain scores at 2 h postoperatively the predefined primary outcome along with delayed need for rescue analgesia and markedly reduced total opioid consumption within the first 24 h. These findings support the efficacy of multimodal strategies in enhancing early postoperative pain control while minimizing opioid exposure. These results were further supported by secondary outcomes, including lower sedation scores, reduced incidence of PONV, and prolonged time to first rescue analgesia in the multimodal group. Importantly, the observed opioid-sparing effect did not compromise analgesic efficacy or patient safety. The discussion below interprets these findings in light of existing literature, explores potential physiological mechanisms, and addresses the clinical implications and limitations of the study.

Effective postoperative pain management is crucial for ensuring optimal recovery and rehabilitation following surgical procedures [24]. In recent years, there has been growing attention toward addressing the challenges of pain control after craniotomy [25]. Given the well-documented adverse effects of opioids, including respiratory depression, nausea, vomiting, and dependency, there has been an increasing emphasis on identifying alternative analgesic strategies to minimize opioid use [11].

A key objective of ERAS is to reduce opioid consumption, thereby decreasing postoperative morbidity, lowering healthcare costs, and improving overall patient quality of life. MMA plays a central role in ERAS protocols, offering a balanced approach to pain control while minimizing opioid-related side effects [26].

Several clinical studies have demonstrated the superiority of opioid-free MMA over opioid-based regimens, showing significant reductions in postoperative complications such as vomiting, opioid consumption, pain scores, respiratory depression, and oxygen dependency [27, 28]. These findings support the continued integration of MMA strategies in postoperative pain management to enhance patient outcomes and recovery.

This prospective, randomized, controlled clinical study, conducted at Zagazig University Hospitals, aimed to evaluate the effectiveness of an opioid-sparing analgesic regime comprising preoperative gabapentin, intraoperative dexmedetomidine, scalp block with bupivacaine at the end of surgery, and postoperative acetaminophen and ketorolac compared to opioid-based analgesia in managing post-craniotomy pain while assessing pain control efficacy and potential adverse effects.

In line with findings from Hinther et al. [24], Ahmad et al. [29], and Lee et al. [30], the present study found no statistically significant differences between the two study groups in terms of preoperative medical and surgical data, including social risk factors (tobacco and alcohol use) and medical comorbidities. This reinforces the consistency of baseline characteristics across similar comparative studies on opioid versus opioid-sparing analgesic protocols.

In the present randomized controlled trial, patients in the multimodal group (Group M) exhibited significantly lower VAS pain scores at 1, 2, and 4 h postoperatively compared to the opioid group (Group O), with the most notable reduction observed at the 2-hour mark the study’s predefined primary endpoint. At this time point, the between-group difference exceeded the minimal clinically important difference (MCID) of 1.0 (*p* = 0.039), indicating both statistical and clinical significance. These findings confirm the superior analgesic efficacy of the multimodal regimen. The early reduction in pain intensity further supports the role of scalp block as an effective regional analgesic technique, in line with previous literature demonstrating the benefits of combining regional and multimodal strategies in post-craniotomy pain control. Accordingly, the null hypothesis assuming no difference in pain scores between groups was rejected. This confirms the superior analgesic efficacy of the multimodal protocol. The clinical value of this difference is reinforced by secondary findings, including delayed time to first rescue analgesia and significantly reduced total opioid consumption in Group M. Together, these outcomes suggest not only better early pain control, but also an opioid-sparing benefit, which is especially relevant in neurosurgical settings where minimizing sedation is crucial for early neurological evaluation. These findings are in line with previous studies. Lee et al. also reported that a change in VAS score exceeding the minimal clinically important difference reflects a meaningful improvement in pain perception. This supports the clinical relevance of the improved postoperative analgesia and reduced opioid requirements observed in our multimodal group [31].

Consistent with our findings, recent literature further supports the effectiveness of similar multimodal approaches in neurosurgical populations. For example, a 2023 randomized trial demonstrated that adding dexmedetomidine to a ropivacaine-based scalp block significantly improved intraoperative hemodynamic stability and reduced perioperative opioid use in elective craniotomy patients. Additionally, scalp block alone has been validated in multiple randomized trials and meta-analyses as an effective technique for reducing early postoperative pain and opioid consumption in this context [32].

Our findings are consistent with prior research focused on post-craniotomy pain management. Zeng et al. demonstrated that preoperative gabapentin administration significantly improved acute postoperative analgesia in craniotomy patients, leading to lower VAS scores and reduced opioid requirements without increasing side effects [33]. Similarly, Sivakumar et al. reported that scheduled IV acetaminophen resulted in significantly reduced pain scores following supratentorial craniotomy when compared to placebo, confirming the value of non-opioid analgesics in this population [34]. Moreover, Duda et al. conducted a recent systematic review and meta-analysis of randomized controlled trials, concluding that scalp block was associated with superior postoperative pain control and opioid-sparing benefits after craniotomy [35]. Collectively, these findings reinforce the validity of implementing MMA strategies specifically within the neurosurgical setting, particularly when opioid minimization and preservation of neurological assessment are critical priorities.

The protocol tested comprising preoperative gabapentin, intraoperative dexmedetomidine, postoperative scalp block, and scheduled non-opioid analgesics fits seamlessly into a neurosurgical ERAS framework. This structured approach enhances early pain control, minimizes opioid exposure, and supports early neurological assessment and ICU discharge readiness. Implementing such a multimodal regimen in elective craniotomy pathways can reduce ICU stay duration and opioid-induced side effects, promoting smoother postoperative recovery While the gabapentin component in our protocol is practical for elective surgeries, its application in emergency neurosurgical cases may be restricted by urgency and patient cooperation. Future adaptations could explore intraoperative or postoperative gabapentin dosing or alternative non-opioid agents suitable for time-sensitive neurosurgical contexts.

A statistically significant difference was observed between the two groups concerning HR at 6, 8, 10, 12, 18, and 24 h postoperatively, with Group M exhibiting higher HR values compared to Group O. Notably, no significant differences in HR were detected during the first 4 h, and this delayed divergence in HR was not accompanied by higher pain scores or increased rescue opioid consumption in Group M. Therefore, it is unlikely that the elevated HR reflects inadequate analgesia. A more plausible explanation may involve the waning sympatholytic effect of intraoperative dexmedetomidine, a known α−2 adrenergic agonist that reduces sympathetic tone and HR during and shortly after administration. As its effects diminish postoperatively, a rebound in sympathetic activity or inter-individual variability in autonomic response may have contributed to the observed hemodynamic pattern. Additionally, MAP was significantly lower in Group M at 2, 4, and 6 h postoperatively, likely due to the combined sedative and hypotensive effects of dexmedetomidine and the analgesic action of the scalp block. Together, these factors may have influenced early postoperative hemodynamic stability in the multimodal group.

The dura mater, a highly vascularized structure, is innervated by the meningeal branches of the trigeminal and vagus nerves, as well as the upper cervical nerves. During craniotomy, routine incision and coagulation of the dura trigger inflammation, leading to intense postoperative pain [36]. The present finding suggests that anti-inflammatory regimens, such as NSAIDs, may be particularly effective for managing post-craniotomy pain. By reducing inflammation and subsequently attenuating the initiation of nociceptive signaling, NSAIDs contributed to the lower pain scores and reduced opioid consumption observed in the multimodal group. Although the use of ketorolac in neurosurgical settings has historically raised concerns due to potential bleeding risks, emerging evidence supports its safety when administered judiciously. Recent studies have shown that ketorolac use in both pediatric and adult neurosurgical populations is not associated with an increased incidence of postoperative hemorrhagic complications [37]. In our study, ketorolac was used at low doses as part of a short-course analgesic regimen, and no bleeding-related adverse events were reported. These findings support the cautious but safe integration of NSAIDs particularly ketorolac into multimodal analgesic protocols for neurosurgical patients, provided that appropriate perioperative monitoring is maintained. In the present study, a statistically significant difference was observed between the two groups in terms of time to first rescue analgesia, total rescue morphine requirements, and overall cumulative opioid consumption. These findings underscore the potential benefits of MMA in reducing opioid consumption and extending the duration of effective pain control. Similarly, Rafiq et al. reported that patients receiving an opioid-sparing multimodal regimen after cardiac surgery required a significantly lower median morphine dose (16 mg) compared to those managed with a conventional morphine-acetaminophen protocol (21.6 mg), reinforcing the opioid-sparing advantage of MMA strategies [38].

Although opioids are highly effective for somatic pain, they are frequently associated with adverse effects, including urinary retention, constipation, nausea, vomiting, respiratory depression, sedation, and confusion. Notably, these effects can increase ICP, potentially mimicking neurological deficits and complicating postoperative assessment [39]. A statistically significant difference was observed between the groups regarding sedation scores at 30 min, 1, 2, 4, 8, 16, and 24 h postoperatively, with group M showing consistently lower sedation levels. This decrease in sedation is likely due to the lack of opioid-induced sedative effects in the opioid-sparing regimen. These results are consistent with the findings of McNicol and Ferguson, who emphasized that while opioid-based regimens can provide effective postoperative pain relief, they are often accompanied by increased rates of sedation and other adverse effects. This underscores the importance of balancing analgesic efficacy with the side-effect profile an objective that MMA protocols aim to achieve [40].

A significant difference was observed between the groups regarding the incidence and frequency of vomiting, with a notably higher occurrence in the opioid-based regimen. Additionally, adverse effects such as pruritus (10%), respiratory depression (10%), and bronchospasm (6.7%) were reported exclusively in the opioid group (Group O), whereas these complications were absent in the multimodal group. These results are consistent with the findings of Jildeh et al., who conducted a randomized controlled trial comparing opioid-based and multimodal non-opioid regimens after arthroscopic shoulder labral surgery. Their study showed that patients receiving opioids experienced side effects such as constipation (1.8 days), nausea (0.2 days), diarrhea (0.2 days), upset stomach (0.2 days), drowsiness (2.4 days), and dizziness (0.4 days). In contrast, those managed with a multimodal regimen reported reduced durations of drowsiness (1.6 days) and nausea (0.1 days). Interestingly, vomiting was not documented in either group [41].

Although the exact etiology of vomiting in the current study remains unclear, its significantly higher incidence in the opioid group aligns with Rafiq et al., who found that no patients in the multimodal group experienced nausea or vomiting, while 13 patients in the opioid group reported these symptoms [38]. Similarly, Ibrahim et al. reported significantly higher postoperative complications in the opioid group, including hypoxia (15%), nausea (60%), shivering (60%), and vomiting (40%), whereas in the multimodal group, hypoxia occurred in only 12%, and shivering in 4.3% [42].

Consistent with these findings, Suzan et al. demonstrated that opioid-free analgesia combined with thoracic epidural anesthesia resulted in a lower incidence of nausea and vomiting, with hypotension suggested as a possible contributing factor [43]. Similarly, Abu Elwafa et al. found that nausea, vomiting, pruritus, sedation, and respiratory depression were significantly lower in the multimodal group compared to the opioid group [44]. While numerous studies support the opioid-sparing benefits of MMA, Mota et al. found no significant differences between multimodal and opioid groups regarding nausea, vomiting, pruritus, or respiratory depression [45]. Similarly, Soltanzadeh et al. reported that the incidence of nausea, vomiting, and respiratory depression within 24 h was comparable between both groups [46]. Further supporting these findings, Özmen et al. noted no statistically significant difference between the groups concerning nausea or vomiting severity [47], while Imantalab et al. and Xiaoxi et al. also reported no significant differences in nausea, vomiting, or hypoxia between the opioid and multimodal groups [48, 49].

No cases of respiratory depression, pruritus, or bronchospasm were observed in the multimodal group, highlighting its favorable safety profile. These findings align with those of Qu et al., who evaluated a neurosurgical ERAS protocol for postoperative pain control following elective craniotomy. Their results demonstrated that none of the patients experienced significant increases in ICP, required surgical revision, showed mental status changes, or needed emergent imaging further underscoring the safety and effectiveness of multimodal pain management approaches [47].

Although Group M achieved superior analgesic outcomes including lower pain scores, fewer opioid-related side effects, and reduced total opioid use excellent satisfaction ratings were paradoxically higher in Group O (36.7%) compared to Group M (13.3%). Given the double-blind design of the study, this difference is unlikely to be attributed to treatment awareness. Instead, it may reflect subjective factors such as patient expectations, perceived intensity of pain relief, or a psychological preference for the sedative effects commonly associated with opioid use. This finding contrasts with previous reports such as Ibrahim et al. [39], who observed higher satisfaction levels among patients receiving MMA. The discrepancy highlights the complex interplay between objective analgesic outcomes and patient-perceived satisfaction, especially in neurosurgical settings.

### Methodological and statistical limitations

This study has several important limitations. First, it was conducted at a single institution, which may limit the generalizability of the findings. Although the sample size was adequately powered for the primary outcome, its modest size restricts the robustness of subgroup analyses. Additionally, the short follow-up period precluded evaluation of long-term neurological or functional outcomes. From a methodological perspective, simple randomization produced balanced groups, yet it does not guarantee equal distribution in smaller trials. Future studies may benefit from block or stratified randomization to improve allocation balance. Moreover, although VAS scores were collected at multiple postoperative time points (e.g., 1 h, 4 h, etc.), only the 2-hour score was predefined for hypothesis testing and sample size calculation. Other comparisons were exploratory, and no adjustment for multiple testing (e.g., Bonferroni or Holm correction) was applied raising the possibility of Type I error. Furthermore, univariate methods were used to analyze repeated outcomes across time, without accounting for within-subject variability. Repeated-measures techniques such as mixed models or MANOVA might have provided a more detailed understanding of pain trajectory over time.

Although morphine consumption was analyzed in this study, it was not the predefined primary outcome. The primary endpoint, used for power calculation, was the VAS score at 2 h postoperatively. Morphine consumption served as a secondary outcome to reflect the potential opioid-sparing effect of the multimodal protocol. However, interpretation should consider the inherent differences in treatment design: the opioid group received scheduled morphine alone, while the multimodal group received multiple synergistic interventions. Therefore, morphine consumption reflects both analgesic efficacy and the nature of the treatment protocol, limiting direct comparability.

### Protocol design and clinical limitations

The multimodal regimen incorporated gabapentin, dexmedetomidine, acetaminophen, ketorolac, and scalp block, while the control group received scheduled IV morphine in alignment with institutional standard of care at the time. This protocol-level comparison reflects common practice in ERAS-based studies, aiming to evaluate the effectiveness of entire analgesic strategies rather than isolate individual drug effects. However, we acknowledge that this approach limits the ability to attribute observed differences to specific components within the regimen. Future trials using factorial or component-based designs are recommended to determine which elements are most essential, synergistic, or potentially redundant. Additionally, the absence of depth-of-anesthesia monitoring tools (e.g., BIS or SedLine) may have introduced some variability in intraoperative sedation. Although TOF and clinical signs were used to guide anesthesia depth, these measures are less precise. Minor differences in extubation timing, though minimized by excluding patients intubated for > 1 h, could have influenced early outcome assessments. Although the control group received opioid-only postoperative analgesia, this regimen reflected the institutional standard of care during the study period and was approved by the institutional ethics committee. All patients were monitored closely and received rescue morphine as needed based on predefined VAS criteria (VAS ≥ 4). No patient experienced undertreated pain. Nonetheless, as multimodal analgesia is increasingly recognized as best practice, future trials should consider enriched control arms to ensure ethical equipoise.

### Future directions

Future investigations should aim to address the current study’s limitations while advancing the clinical applicability of MMA in neurosurgery. Multicenter randomized trials with larger and more diverse patient populations are needed to improve external validity and enable robust subgroup analyses. Employing block or stratified randomization could further enhance group balance, especially in trials with limited sample sizes. Moreover, factorial or component-based study designs are recommended to isolate the specific contributions of individual protocol elements such as gabapentin, dexmedetomidine, scalp block, or NSAIDs toward analgesic efficacy and safety. This would facilitate streamlined regimens, reduce pharmacologic burden, and optimize resource utilization. Long-term follow-up studies should also be conducted to evaluate neurological, cognitive, and functional outcomes associated with MMA strategies. In parallel, cost-effectiveness analyses are warranted to assess the feasibility of implementing such protocols in routine neurosurgical practice. Finally, integrating objective depth-of-anesthesia monitoring tools (e.g., BIS or SedLine) into future protocols may improve intraoperative consistency and allow for more precise assessment of anesthesia-related effects on postoperative recovery.

## Conclusion

This study demonstrates that opioid-sparing MMA comprising preoperative gabapentin, intraoperative dexmedetomidine, and a scalp block with bupivacaine administered at the end of surgery, alongside scheduled postoperative acetaminophen and NSAIDs provides effective pain relief with a delayed need for rescue analgesia and reduced total opioid consumption compared to opioid-based regimens. Furthermore, the incidence of nausea and vomiting was significantly lower in the multimodal group, whereas sedation was notably higher in the opioid group. These findings suggest that opioid-sparing MMA offers a safer and more patient-centered approach to pain management, potentially serving as a superior alternative to traditional opioid-based strategies in postoperative care.

## Supplementary Information

Supplementary Material 1.

## Data Availability

The datasets generated and analyzed during the current study are not publicly available but are available from the corresponding author upon reasonable request.

## Abbreviations

CT: Computed tomography
ICP: Intracranial pressure
NSAIDs: Non-steroidal anti-inflammatory drugs
ICU: Intensive care unit
ASA: American Society of Anesthesiologists
GCS: Glasgow coma scale
IRB: Institutional review board
BMI: Body mass index
ECG: Electrocardiogram
CBC: Complete blood count
INR: International normalized ratio
HIV: Human immunodeficiency virus
HCV Ab: Hepatitis C virus antibodies
HBV Ag: Hepatitis B surface antigen
Ab: Antibody
MRI: Magnetic resonance imaging
VAS: Visual analogue scale
IV: Intravenous
HR: Heart rate
BP: Blood pressure
PNS: Peripheral nerve stimulator
TOF: Train-of-four
NMBA: Neuromuscular blocking agent
ETCO2: End tidal CO2
CVL: Central venous line
PONV: Post operative nausea and vomiting
MMA: Multimodal analgesia
MAP: Mean arterial pressure
OFA: Opioid free analgesia
ERAS: Enhanced recovery after surgery
